# Putrescine treatment has a higher effect on 5mC DNA methylation profile of wheat leaves under white than under blue light conditions

**DOI:** 10.1038/s41598-025-08184-y

**Published:** 2025-07-02

**Authors:** Imre Majláth, Kinga Benczúr, Altafur Rahman, Tibor Janda, István Likó, János Kádas, Magda Pál

**Affiliations:** 1 Hungarian Research Network, Centre for Agricultural Research, Agricultural Institute, Brunszvik 2, Martonvásár, 2462 Hungary; 2 Department of Plant Physiology and Ecology, Institute of Agronomy, MATE, Villányi 29-43, Budapest, 1118 Hungary; 3 UD-GenoMed Medical Genomic Technologies Ltd, Nagyerdei Krt. 98, Debrecen, 4032 Hungary

**Keywords:** DNA methylation, Light quality, Polyamine, Metabolite profile, Wheat, Molecular biology, Plant sciences

## Abstract

Polyamines play a pivotal role in plant growth and development by modulating primary metabolism and gene expression patterns. However, their regulatory effect on epigenetics is still not clear. The impact of exogenous putrescine in wheat leaves was investigated to reveal novel insights into the intricate relationship between polyamines and DNA methylation. Since the outcome of putrescine treatment may vary depending on the light quality, the experiment was performed under both white and blue light, and MeDIP-seq method was applied to reveal the global methylation profile. Metabolite profiling revealed distinct changes induced by blue light and/or putrescine, and that the effect of putrescine in most cases was dominant under blue light. Blue light also exerted strong effects on methylation, and putrescine application could slightly induce further changes. Putrescine had a higher impact under white light, namely parallel with increased leaf spermidine level, a lower level of up-methylated genes involved in cellular component, but a higher level of down-methylated genes involved in molecular function and biological processes occurred compared to the blue light. Our results demonstrated that putrescine excess has a regulatory role in DNA methylation. These findings are also useful in understanding the relationship between polyamine metabolism and light conditions.

**Supplementary Information:**

The online version contains supplementary material available at 10.1038/s41598-025-08184-y.

## Introduction

Plants are sessile organisms exposed to very changeable and often unfavourable growth conditions. Thus, plants depend highly on gene expression and metabolite changes to respond quickly and appropriately to environmental stimuli. Polyamines (PAs) are well-studied plant growth regulators due to their participation in the organisation of several plant growth and development processes and stress tolerance in plants. Both increased endogenous synthesis and exogenous supply of PAs have been demonstrated to protect against different abiotic stress factors^1^. The most abundant PAs are putrescine (PUT), spermidine (SPD) and spermine (SPM), and the latter two are formed due to the subsequent addition of an aminopropyl moiety to the PUT skeleton. Their metabolism is very dynamic, partly because they are synthesised from each other, and on the other hand, due to the back-conversion of SPM/SPD to PUT. Together is the so-called PA-cycle^2^. It is more and more evident that the balance between the positive and negative effects of PAs is influenced by the fine-tuning of PA metabolism, which in turn plays an important role in the adaptation of plants. The metabolic pathways of polyamines (PAs) are intricately linked to the biosynthesis of various plant hormones and bioactive molecules, including ethylene, γ-aminobutyric acid (GABA), hydrogen peroxide (H_2_O_2_), proline and phytochelatins^2^. However, the biosynthesis of PAs is also linked to the methylation of genes. S-adenosylmethionine (SAM) is the common precursor of the synthesis of higher PAs (SPD and SPM) and DNA cytosine methylation^3^. The aminopropyl group of decarboxylated S-adenosylmethionine (dcSAM), which is converted from SAM, is added to PUT or SPD to form SPD or SPM, while the transfer of methyl group from SAM to cytosine at the C-5 position yields 5-methylcytosine (5mC).

Chromatin-based regulation without changing the DNA sequence plays an important role in the adaptation mechanism^4^. Epigenetic processes can easily regulate metastable changes at the gene expression level, and the resulting fine-tuned gene expression patterns can be responsible for observed phenotypic differentiation and adaptation to specific environments during the lifetime of plants^5–7^. DNA methylation is an essential epigenetic process which plays a fundamental role in gene regulation^8^. The most widely and extensively studied DNA methylation in eukaryotes is the methylation of the fifth position of the cytosine catalysed by DNA methyltransferase. Methylated DNA co-immunoprecipitation sequencing (MeDIP-seq) detects 5mC exclusively, targets low CpG density regions and finds high methylated regions in the whole genome rapidly and accurately^9^. While the relation between elevated PA uptake and DNA methylation has been extensively examined in humans and animals^3^, corresponding investigations in the realm of plant biology remain scarce. In mature wheat embryo culture, it was found that SPD levels correlated with DNA hypermethylation, and SPM levels with DNA hypomethylation^10^. In *Cattleya tigrina,* low SPD content was related to DNA methylation; in addition, higher DNA methylation percentage was detected in those samples where the level of PUT was lower^11^. In cabbage plants, seed-soaking with PUT resulted in demethylation both under NaCl stress and stressless conditions^12^. According to these findings, it could be hypothesised that as increased SPD and SPM synthesis reduce PUT and dcSAM levels, and as the competitive inhibitory effect of dcSAM on DNA methyltransferase has been suggested, the reduced dcSAM level can result in higher DNA methylation^13,14^.

Light is the most critical factor in plant growth and acclimation. Blue ratio of solar radiation varies during the year and even during the day due to certain environmental factors, for example, cloud cover^15^. Blue light is known to suppress plant growth extension, resulting in a compact phenotypic appearance compared to plants grown without blue light. However, there is growing evidence that blue light has beneficial effects, for example, enhance yield, increase the antioxidant capacity, the levels of protective metabolites, and improve plant tolerance^16,17^. Light-induced changes have been reported in various plant species, where not only the light quantity, but the quality also modulates gene expression pattern and metabolite composition^18–22^. The development of light-emitting diode (LED) technology opens new possibilities in plant biology to reveal the detailed effects of manipulated light characteristics and optimise the regulation of plant physiology. Light quality exerts a significant influence on the endogenous concentrations of plant growth regulators, including phytohormones and PAs^18^. Previously, it has been reviewed that PA metabolism is modulated by both the light quantity and quality in different plant species^2^. In addition, the length of the daily light period and the hours of illumination also influenced it^23^. Thereby light can modulate the physiological functions of PAs and PA-induced protective responses. Also in wheat, although changes in light quantity induced pronounced changes in the endogenous PA pool, hardly any influence was detected in the PA metabolism at the gene expression level. In addition, exogenous PA treatments influenced the PA metabolism differently under altered light intensities, leading to successful adaptation to lower light conditions^24^. In contrast, different light spectral conditions did not cause pronounced changes in the PA pool, but blue light decreased and red/far-red light increased the gene expression of PA metabolism-related genes. Roborative effects of PA treatments were also detected, especially under blue light^25^. Blue light-induced alterations in metabolite composition, including plant pigments, thiols, and amino acids, have been documented in wheat plants^26^. Furthermore, blue light-mediated regulation of gene expression and metabolite profiles has been demonstrated to have the potential to enhance freezing tolerance, exemplified by studies in barley^27^. In our recent study, it was demonstrated that PUT pre-treatment at 0.5 mM concentration, after 7 days had a protective effect against cadmium (Cd) stress in young wheat plants under both white and blue light conditions. In addition, blue light-regulated Cd tolerance was modified by PUT excess, regarding the phytochelatin synthesis, PA metabolism, and accumulation of phenolic compounds and plant hormones^28^.

PAs are promising biostimulants. Previously, it has been revealed that PUT treatment has roborative and protective effects in wheat plants, in addition close relationship between PA metabolism and light quantity exists. Thus, light spectra may influence the outcome of the PA treatment. The main goal of our study was to investigate further the background of the beneficial effect of PUT treatment and highlight the differences in response to PUT supplementation under white and blue light conditions in wheat seedlings. Both light and PUT may exert their effects at both metabolite and gene expression levels. In addition, due to the SAM as a common precursor, the synthesis of SPD and SPM, and DNA methylation is overlap, thus we hypothesised that PUT induce epigenetic changes. The present study is the first to investigate the interaction of blue light and PUT treatment at the DNA methylation level using MeDIP-seq method in wheat plants. Changes in endogenous PA levels, together with changes in the levels of certain metabolites, were monitored after 7 days of 0.5 mM PUT treatment.

## Materials and methods

### Plant material, growth conditions and treatments

Seeds of winter wheat plants (*Triticum aestivum* L. variety ‘Mv Béres’) were germinated for 3 days at 26 °C in the dark. Fifteen seedlings were planted into each plastic pots (volume: 0.4 L) and grown on modified Hoagland solution in Conviron PGR-15 plant growth chambers (Controlled Environments Ltd., Winnipeg, Canada) under 16/8-h light/dark periodicity and 22/20 °C day/night temperature with and 75% relative humidity^29^, and under two different light spectrums. Under white (W) light, 32.32% blue, 45.71% green, 20.71% red and 1.25% far-red composition, while under blue (B) light, 82.14% blue, 1% green, 16.7% red and 0.17% far-red composition was applied at 250 µmol m^−2^ s^−1^ photosynthetic photon flux density (PPFD) (Table S1). Light spectral conditions were established using modules equipped with LEDs according to a previous study^26^.

Seven days after the germination, half of the plants grown under either W or B light conditions were further grown without any treatment (WC or BC), while the other part of them got 0.5 mM PUT supplementation in the hydroponic solution (WP or BP) in the next 7 days. The PUT concentration and the duration of the treatment were selected according to our previous experiments on wheat seedlings^24,25^, where it was found under different light conditions (light intensities and spectral compositions), that 0.5 mM PUT treatment had positive effects and compensate physiological changes induced by the not favorable light conditions. In addition to these, recently we also demonstrated that 0.5 mM PUT treatment for 7 days as a pre-treatment elevated Cd stress under both white and blue light conditions^28^. During the experiment, the nutrition solution was changed every 2 days. At the end of the treatments, the leaves and the roots samples were collected for further analysis.

### Sample preparation and GC metabolomics analyses

Sample preparation was performed based on Gondor et al.^24^. Samples were extracted in methanol solutions (60% and 90%) and an internal standard was added before the extraction. For derivatization, an aliquot from the supernatant was dried under vacuum. The samples were injected into LECO Pegasus 4D GCxGC TOFMS (LECO Corporation, St Joseph, Michigan, USA), where the primary column was Rxi-5MS (30 m, 0.25 mm ID, 0.25 µm)(Restek GmbH, Germany), while the secondary column was Rxi-17Sil MS (1.5 m, 0.15 mm ID, 0.15 µm)(Restek GmbH, Germany). Identification was based on the Kovats retention index, the NIST Mass Spectral Search Program (InChI Library v 1.05) and Leco-FiehnLib for non-targeted assessments, while standards for targeted assessments. For data evaluation, LECO ChromaTOF 4.72 was used. The unit of measurement in the case of targeted compounds is µg g^−1^ FW. Equations for calibration and correlation coefficients targeted compounds listed in (Table S2).

### Analysis of PUT, cadaverine (CAD), SPD, SPM and DAP

200 mg of plant tissue was homogenised in 2 ml 0.2 N HClO_4_, and the homogenates were centrifuged at 4 °C for 10 min at 10,000 g. The supernatants were used for dansyl chloride derivatisation reactions according to Németh et al.^30^. Briefly, 100 μl aliquots of the supernatant were added to 200 µl of saturated sodium carbonate and the reaction was started with 400 μl of dansyl chloride in acetone (5 mg ml^−1^). The mixture was incubated at 60 °C for 1 h in the dark, and stopped with 100 μl of proline (100 mg ml^−1^). After 30 min, the PAs were extracted with 500 μl of toluene with vigorous vortexing for 30 s. The organic, upper phase, containing the PAs, was completely dried under nitrogen. The PA residue was dissolved in 1 ml of methanol, ultrafiltered through nylon membranes and used for analysis. The analysis of dansylated PAs was carried out according to Pál et al.^25^ via HPLC using a W2690 separation module on a reverse phase column (Kinetex C18, 5 μ, 100 × 4.6 mm, Phenomenex, Inc.) and a W474 scanning fluorescence detector with excitation at 340 nm and emission at 515 nm (Waters, Milford, MA, USA).

### MeDIP-sequencing

DNA isolation, quality control of the DNA isolates, methylated DNA Immunoprecipitation, next-generation MeDIP DNA sequencing, and the bioinformatical analysis of the primary results were performed at the UD-GenoMed Medical Genomic Technologies Ltd., Debrecen, Hungary.

#### DNA isolation and DNA QC measurements

The genomic DNA samples were isolated with NucleoSpin^®^ Plant II Kit (MACHEREY-NAGEL GmbH, Düren, Germany) according to the manufacturer’s instructions. This kit is designed for the isolation of genomic DNA from plant tissue using two optimized lysis buffer systems based on the established CTAB and SDS methods. DNA extracts were stored at − 80 °C until sequenced. Before the isolation for plant tissue homogenization green leaves were ground with mortar and pestle in the presence of liquid nitrogen.

Quantity and quality of the purified DNAs were checked with photometric method and 1% agarose electrophoresis. The DNA concentration and A260/280 ratio were measured with DeNovix spectrophotometer (DeNovix, Inc., Wilmington, DE, USA) according to the manufacturers’ instructions.

#### DNA fragmentation and methylated DNA immunoprecipitation

For immunoprecipitating the methylated DNA from the plant DNA isolates, a methylated DNA Immunoprecipitation (MeDIP) Kit (Abcam, Ltd., Cambridge, UK) was used. 1 ug of each DNA isolates added to the reaction buffer and sheared by sonication. DNA sonicated 2 × 5 pulses of 30 s at high energy level using Bioruptor Plus sonication device (Diagenode, LLC., Denville, NJ, USA) followed by 30 s rest on ice between each pulse and finally sonicated DNA incubated at 95 °C for 2 min and placed on ice. The fragmented DNA measured by Bioanalyzer 2100 (Agilent Technologies, Inc., Santa Clara, CA, USA) instrument using Agilent DNA 1000 Kit (Agilent Technologies, Inc., Santa Clara, CA, USA) according to manufacturer’s protocol to verify the size distribution of sonicated DNA. The next steps of immunoprecipitation procedure were done according to the manufacturers’ instructions.

#### MeDIP sequencing library generation and NGS library validation

To obtain the sequencing data high throughput sequencing analysis was performed on Illumina (Illumina, Inc., San Diego, CA, United States) sequencing platform. The DNA sequencing library was prepared using immunoprecipitated (12 samples) and pooled, fragmented IP input DNAs (4 pooled samples) with xGen™ ssDNA & Low-Input DNA Library Preparation Kit (Integrated DNA Technologies (IDT), Inc., Coralville, IA, USA) according to the manufacturer’s instructions.

Final libraries were measured using Bioanalyzer 2100 (Agilent Technologies, Inc., Santa Clara, CA, USA) instruments using Agilent DNA 1000 Kit (Agilent Technologies, Inc., Santa Clara, CA, USA) according to manufacturer’s protocol to verify the size and validate the quality.

#### Next generation sequencing and pre-processing of DNA-seq data

The normalized, pooled, and denatured library samples were sequenced on the Illumina NextSeq platform (Illumina, Inc., San Diego, CA, United States) using 75 + 75 bp paired-end chemistry with NextSeq 500/550 HiOutput Kit v2.5 (150 Cycles), producing on average 25 million raw reads per sample.

Bioinformatics analysis of differential methylation experiment from MeDIP was performed after the MeDIP paired end sequencing data were exported in FASTQ file format. The raw data from the sequencer, the reads collected in the fastq file (short sequencing reads), were subjected to qualitative analysis using the Fastqc (Babraham Bioinformatics) program.

The reads were trimmed using Trim Galore and Cutadapt to remove bases where the PHRED quality value was less than 20^31^. Trimmed sequences were removed if they become shorter than 50 bases. The FASTQC program (https://www.bioinformatics.babraham.ac.uk/projects/fastqc/) was used to evaluate the qualities of original and trimmed reads.

#### Sequence alignment and primary data analysis

The trimmed sequence sets (FASTQ-formatted) were aligned to reference genome using Burrows‐Wheeler Aligner (BWA)^32^. The wheat reference genome sequence in fasta and the annotation gff format were downloaded from the NBCI website (Representative genome: *Triticum aestivum* (bread wheat) (assembly GCF_018294505.1_IWGSC_CS_RefSeq_v2.1_genomic.fna)).

The analysis of the enriched methylated DNA locus was performed with bedtools (https://bedtools.readthedocs.io/en/latest/) and R statistical functions. The position and number of reads matching the wheat genome are characteristic of the methylation of the given DNA section. Therefore, using the bedtools program, the coordinates of the covered DNA sections (loci) and then the number of reads corresponding to these loci for each sample were determined. The Bioconductor package edgeR were used for differential methylation analyses of read counts arising from MeDIP. The package can be applied to any technology that produces read counts for genomic features. Testing for differential methylation of the locus performed by the GLM likelihood ratio test based on the idea of fitting negative binomial GLMs with the Cox-Reid dispersion estimates. Three independent biological repetitions were used for each treatment group.

### Overlap analysis between genomic ranges

The sequencing resulted in 10,969,813 differentially methylated raw read counts. To filter out counts with low-level methylation, the following criteria was applied. Two samples which have a read count greater than ten in any group were considered differentially methylated locus. The clean read counts with *p* < 0.01 were mapped to the IWGSC CS RefSeq v2.1 reference genome to find the overlaps between the methylated loci and the reference genomic regions. Before the overlap matching analysis, the start coordinates in the reference genome were extended to quasi promoter regions with 3 kb sequences on each strand, using the Biocmanager^33^, rtracklayer^34^ and GenomicRanges^35^ packages in *R* programming (v4.1 and 4.3.2.). The number of differentially methylated gene elements (DMGs) was computed for each treatment combinations. The result of the overlap analysis of genomic ranges was further filtered for log_2_FC >|1.5|. The Entrez gene identifiers of the up- and down-methylated datasets were then separately used for downstream analyses. An upset analysis was performed to find and visualise the numbers of common and individual transcripts between the treatment combinations, using the UpSetR package^36^.

### Principal component analysis (PCA) for DNA methylation data

The PCA was computed using the FactoMineR^37^ and FactoExtra^38^ packages in *R*. The biplot illustration shows the magnitude and direction of the coefficients for the original variables in a plane of PC1 and PC2, based on the similarity of individual variable.

### Gene ontology analysis (GO)

The GO mapping of the Entrez IDs was carried out by using the online analysis tool agriGO v2.0^39^. Singular enrichment analysis (SEA) was performed using the ENSEMBL gene IDs of the significant DMGs (log_2_FC >|1.5|) and the *Triticum aestivum* (Chinese Spring) gene ID (GO version 2016) as a background. To compute the significantly overrepresented terms, a hypergeometric statistical test combined with the Hochberg (FDR) multiple test adjustment method (*p* < 0.05) was set to regulate the occurrence of false positives. The ShinyGO v0.80 application^40^ was also run to remove redundant terms from three major GO classes.

### Gene expression analysis

Total RNA was extracted from fully developed leaf samples (approx. 100 mg) and cleaned using TRI Reagent® and Direct-zol™ RNA MiniPrep Kit (Zymo Research, Irvine, CA, USA). cDNA synthesis was carried out by using M-MLV Reverse Transcriptase (Promega Corporation, Madison, WI, USA) and oligo(dT)18 (Thermo Fisher Scientific). PCRBIO SyGreen Mix (PCR Biosystems, London, UK) and CFX96 Touch™ Real-Time PCR Detection System (Bio-Rad, Hercules, CA, USA) were used for quantitative real-time PCR reaction according to Tajti et al.^41^. For primer sequences see Table S3. 42, 43.

Gene expression analysis was performed in order to reveal the relationship between the methylated genome profile and the expression level of certain genes. Genes were chosen according to the detected, most remarkable changes (log_2_FC >|5|) in methylome.

### Statistical analysis

Three independent experiments were performed, and the most representative data are presented here regarding the PA and metabolite contents. This repetition was also used for the methylation profile and gene expression analyses. The results represented at least three biological replicates for each treatment group. The Duncan post-hoc test was performed using SPSS 16.0. 2-way ANOVA test using SPSS 16.0 was performed on the results of PA and metabolite contents.

## Results

### Changes in certain metabolite quantities

Certain metabolite compounds related to the tricarboxylic acid cycle (TCA cycle), amino acid synthesis and PA metabolism were detected in the leaves of plants after the treatments. Exposure to B light or PUT treatment also elicited distinct alterations in the metabolite profile compared to the W light control group (refer to Table 1). Notably, under B light, however not always statistically significant increases were found in asparagine (Asn), serine (Ser), aconitic acid (ACON), itaconic acid (ITA), oxalic acid (OXA), 5-Oxo-proline (Glp), and gamma-aminobutyric acid (GABA), alongside decreases in shikimic acid (SHIK) and malic acid (MAL) levels. Conversely, PUT treatment under W light conditions resulted in the accumulation of aspartic acid (Asp), SHIK, and other tricarboxylic acids (TCA) cycle intermediates, including citric acid (CIT), succinic acid (SUCC), fumaric acid (FUM), and OXA. Intriguingly, following PUT treatment, reduced levels of asparagine (Asn), glycine (Gly), alanine (Ala), ACON, glutamic acid (Glu), Glp, and ornithine (Orn) were observed compared to the W light control conditions. PUT treatment also induced certain changes under B light, which were partly similar to those observed under W light, however sometimes the effect of B light was more dominant. So, PUT treatment could specifically increase the levels of SHIK and OXA, while decreased the Asn, Gly, Glp and Orn contents. The effect of B light was more ascendant even after PUT treatment in the case of ACON, MAL and GABA. Furthermore, an additive effect of B light and PUT treatment resulted in the most elevated concentrations of threonine (Thr), Ser and Glu, accompanied by the lowest levels of phenylalanine (Phe) and MAL observed in plants treated with BP.

**Table 1:**
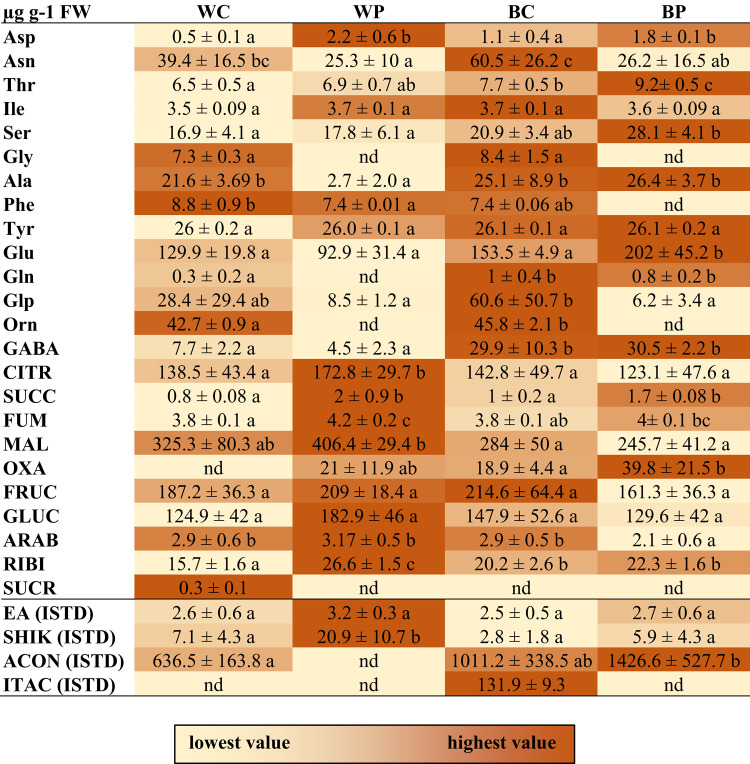
Colour-scaled table shows changes in metabolite profile without (C) or with (P) 0.5 mM putrescine pre-treatment under white (W) or blue (B) light conditions in the leaves of wheat plants.

Values are means ± SD. Statistically significant differences are indicated with different letters at *p* < 0.05 level. nd: under the detection limit. The coloured background was formatted for the individual metabolites; light brown indicates the lowest and dark brown the highest value. *ACON* aconitic acid, *Ala* L-Alanine, *ARAB* D-arabinose, *Asn* asparagine, *Asp* L-aspartic acid, *CITR* citric acid, *EA* ethanolamine, *FUM* fumaric acid, *FRUC* D-fructose, *GABA* gamma-aminobutyric acid, *Gln* L-glutamine, *Glp* L-5-oxoproline, *Glu* L-glutamic acid, *GLUC* D-glucose, *Gly* glycine, *Ile* L-isoleucine, *ITAC* itaconic acid, *MAL* malic acid, *Orn* L-ornithine, *OXA* oxalic acid, *Phe* phenylalanine, *RIBI* d-ribose, *SHIK* shikimic acid, *SUCC* succinic acid, *SUCR* sucrose, *Ser* serine, *Thr* L-threonine, *Tyr* L-tyrosine. ISTD indicate internal standard calibration for non-targeted analysis.

Variance analysis also showed a special, dominant influence of light treatment on Asn, Phe, SUCC, MAL, Glu, GLUC, and ARAB. At the same time, the significant effect of PUT treatment on the investigated parameters was the most pronounced in the case of Asp, Gly, SHIK, CITR, ACON, FUM, OXA, and Orn. The interaction of PUT treatment and light conditions was also confirmed to be significant and additive due to the combined PUT and B light treatment in the case of Ser, CITR, and Glu (Table S4).

### Changes in polyamine contents and spermidine synthase gene expression level

**Fig. 1:**
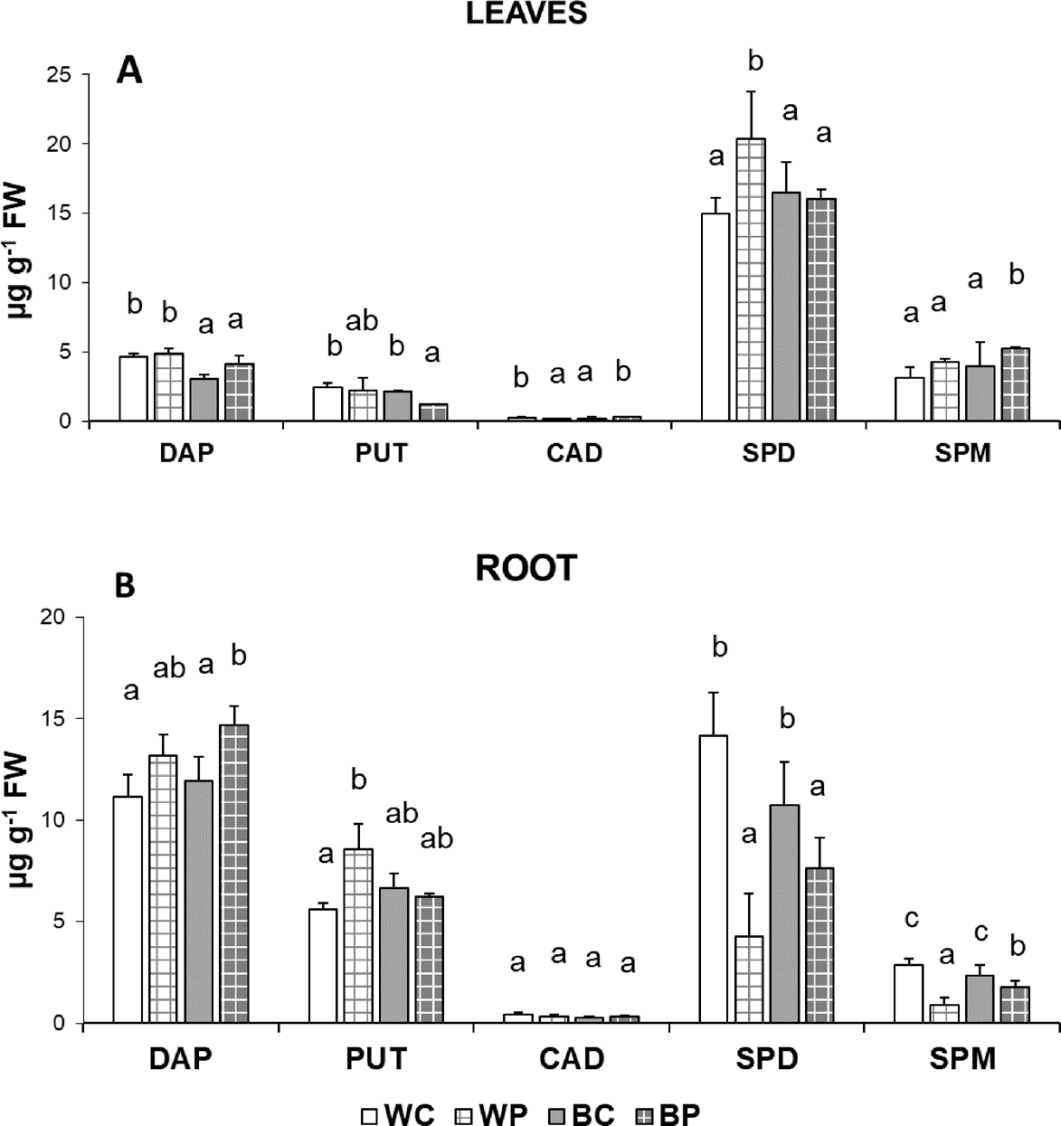
Polyamine contents (DAP: 1,3-diaminopropane, PUT: putrescine, SPD: spermidine and SPM: spermine) without (C) or with (P) 0.5 mM putrescine pre-treatment under white (W) or blue (B) light conditions in the leaves (**A**) and roots (**B**) of wheat plants. Values are means ± SD. Statistically significant differences are indicated with different letters at *p* < 0.05 level.

The most dominant PA was SPD both in the leaves and roots of wheat plants (Fig. 1A–B). B light alone did not induce remarkable differences in PA content either in the leaves or roots. Excess of PUT slightly influenced the PA levels in the leaves, but increased statistically significantly the SPD content under W light. PUT treatment resulted in more characteristic changes in the roots. The catabolite of the SPD and SPM oxidation, DAP increased by PUT treatment, but this increment was statistically significant only under B light. The level of PUT increased after PUT treatment only under W light conditions. On the other hand, the amount of SPD decreased after PUT application under both light conditions. A similar tendency was found in the case of the SPM.

Variance analysis also confirmed that light alone had a significant effect on leaf DAP and SPD content, and root DAP, PUT, SPD and SPM contents, while PUT treatment had a significant, unique effect only on leaf DAP and PUT. In light and PUT treatment interaction significant effect was observed in the case of lead CAD and SPD, and root PUT, SPD and SPM (Table S5).

**Fig. 2:**
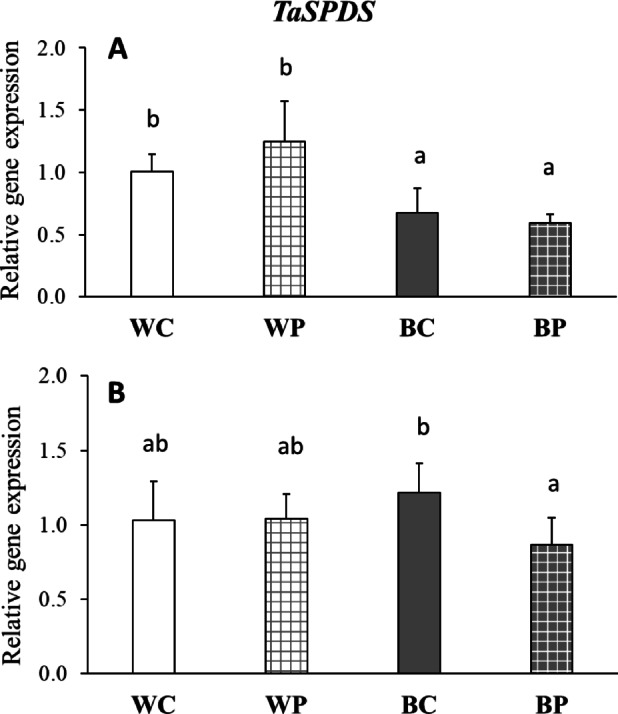
Gene expression level of spermidine synthase (*TaSPDS*) without (C) or with (P) 0.5 mM putrescine pre-treatment under white (W) or blue (B) light conditions in the leaves (**A**) and roots (**B**) of wheat plants. Values are means ± SD. Statistically significant differences are indicated with different letters at *p* < 0.05 level.

Despite the similar levels of leaf SPD content under W and B light conditions, the gene expression level of spermidine synthase (*TaSPDS*) in the leaves was slightly lower under B light both with or without PUT treatment (Fig. 2A). Tendentious increase in SPD content of WP-treated leaf samples compared to the control (WC) was not in relation with the pattern of leaf *TaSPDS* expression in case of these treatments. Although the PUT treatment reduced the SPD content in the roots, it was only able to induce a slight decrease in *TaSPDS* expression under B light (Fig. 2B).

### Analysis of changes in methylome

#### The methylome analysis of wheat under different light and PUT treatments

Filtering out the read counts with low-level methylation from 10,969,813 differentially methylated raw counts, 539,307 high-quality, clean reads counts were obtained, with an average of 19,926 reads for each treatment combination. After the cutoff at *p* < 0.01, 65,441 significant loci were matched to the IWGSC CS RefSeq v2.1 genome, which resulted in 2873 overlaps between the query loci and reference genomic regions (Table S6).

**Fig. 3:**
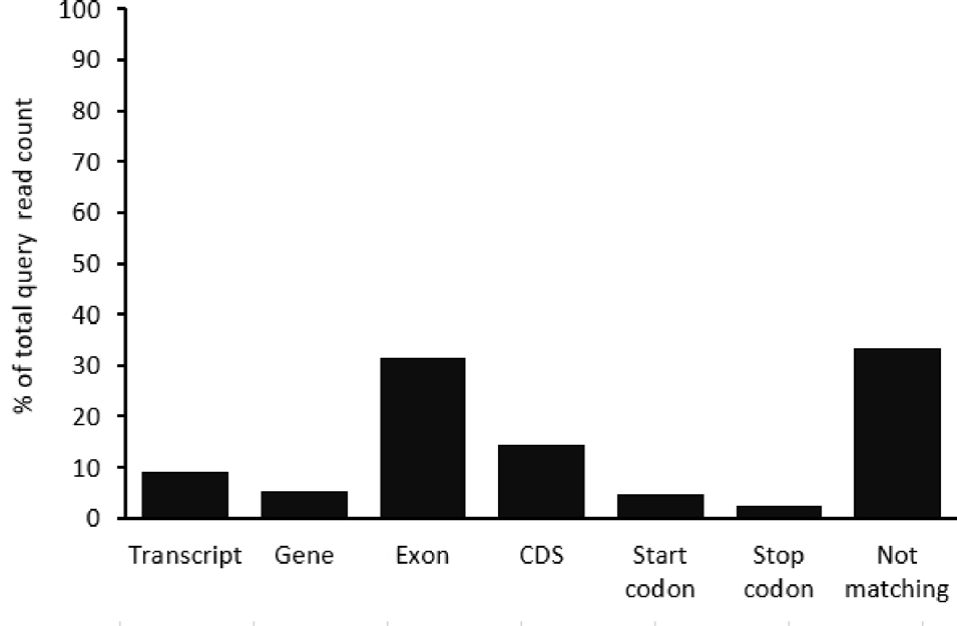
The distribution of the localization of differentially methylated gene elements. Percentages are calculated from the overlapping and the total locus counts of the query genome.

The ratio of differentially methylated elements (transcript, gene, exon, CDS, start codon, stop codon) can be seen in Fig. 3. The 33.25% of the query loci did not match to any of the genetic features. The exon type ratio was 31.5%. The CDS were matched in 14.3% and the representation of the other types was less than 10%.

For ease of interpretation, the comparisons of the original light and PUT treatments (_vs._) were converted into terms as follows: BC versus WC = ’BLUE_effect’, BP versus WC = ’BLUE_and_PUT_effect’, WP versus WC = ’PUT_effect_at_WHITE’, BP versus BC = ’PUT_effect at_BLUE’, WP versus BC = ’WHITE_and_PUT_effect’, WP versus BP = ’WHITE_effect_at_PUT’.

**Fig. 4:**
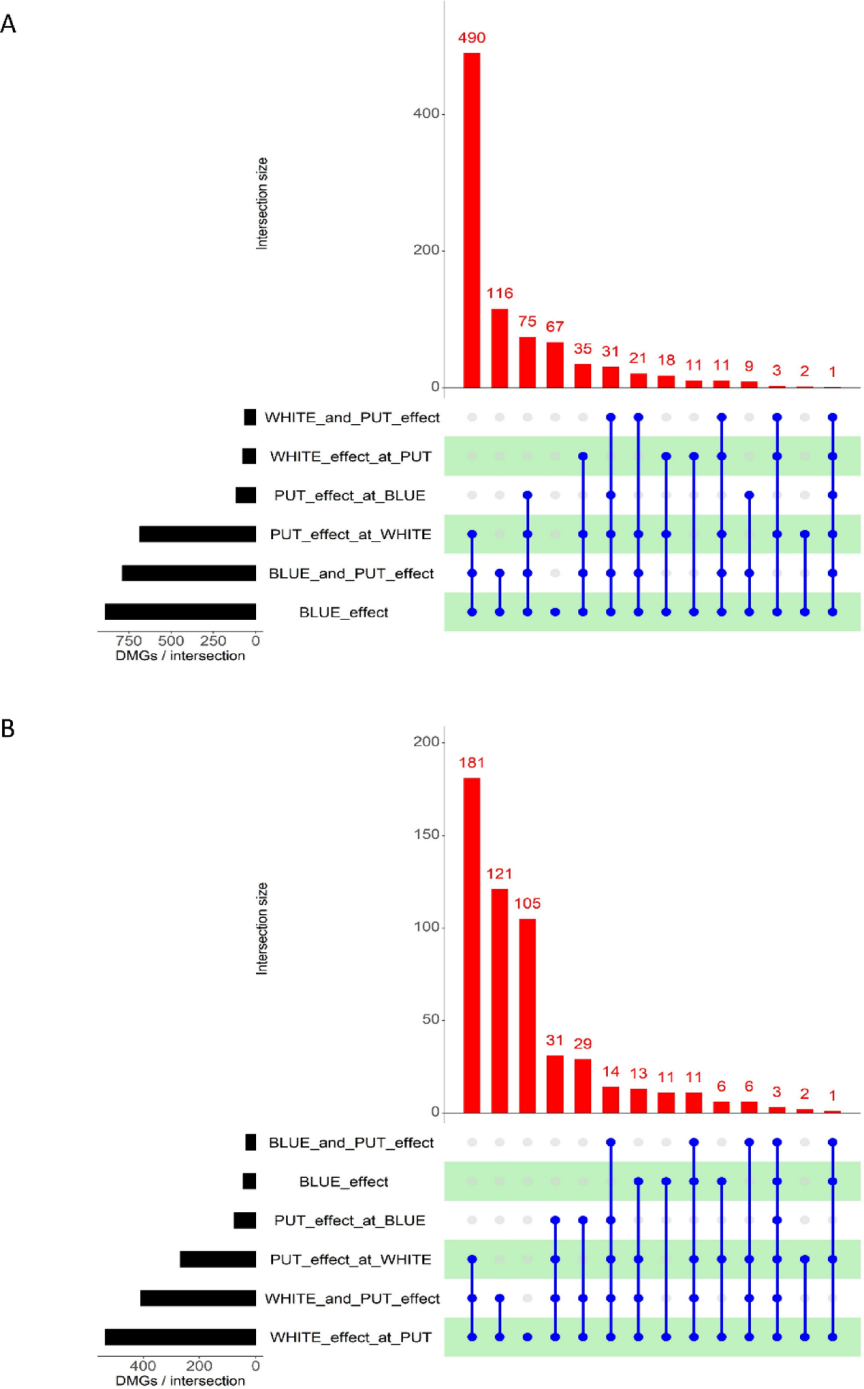
The number of overlaps of differentially methylated genes (DMGs) in cases of up- (**A**) and down-methylation (**B**). The black columns show the sizes of the input sets. The red columns indicate the number of overlaps between the treatment combinations. Blue bars indicate the combination of sets of each intersection.

The numbers of the common and individual differentially methylated genes (DMGs) were different in the various intersections of up- and down-methylated sets. The size of sets was generally higher in the case of up-methylated DMGs, especially at PUT_effect_at_WHITE, BLUE_and_PUT_effect and BLUE_effect. Their intersection included 490 genes (Fig. 4A). The down-methylated set size was high at PUT_effect_at_WHITE, WHITE_and_PUT_effect and WHITE_at_PUT_effect. Their intersection contains 181 DMGs and 121 were also common in the latter two treatments (Fig. 4B). Unique list of DMGs were found only under BLUE_effect and WHITE_effect_at_PUT of the up- and down-methylated dataset, respectively. The common gene was TraesCS5D02G403200 (protein LEO1 homolog isoforms) in the intersection of all sets of the up-methylated DMGs and TraesCS1B02G312000 (V-type proton ATPase subunit E-like), TraesCS5A02G146900 (chalcone isomerase-like protein 2), TraesCS7D02G132900 (uncharacterized protein LOC123165569) genes were in the common set of the down-methylated DMGs.

**Fig. 5:**
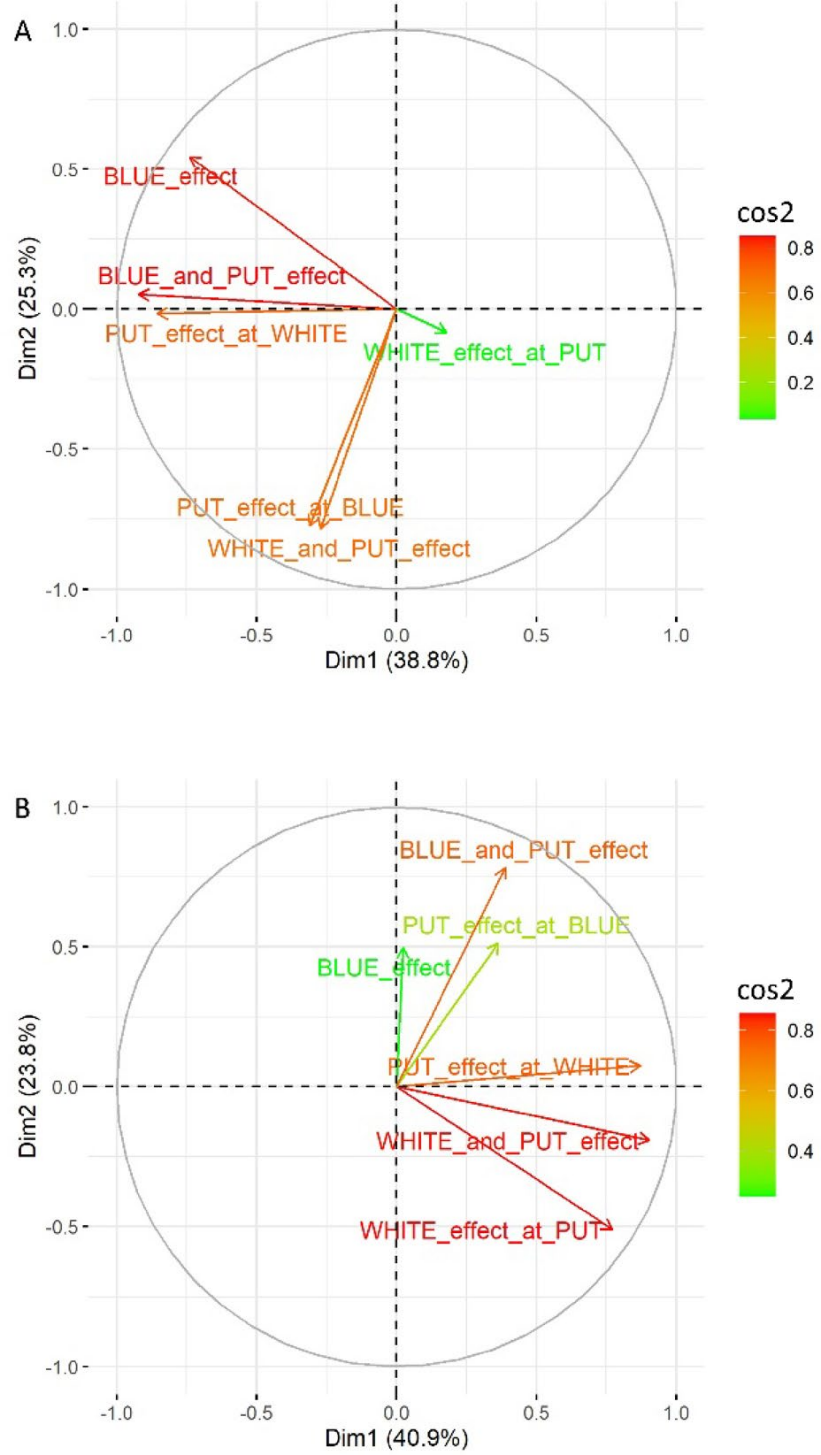
The PCA loading plots show the eigenvalues of the original variables on the PC1 and PC2 planes (Dims) for the up- (**A**) and down-methylated (**B**) datasets, based on the log_2_FC values of the DMGs. Percentages represent the explained variance of the PCs. Cos2 scale means the accuracy of the representation on the PC planes.

Multidimensional scaling of the up- and down-methylated genomes was performed using PCA. The scaling was based on the fold-change values of the DMGs. The PC1 and PC2 represented the explained variance in 38.8% and 25.3% of the up-methylated genome, and 40.9% and 23.8% of the down-methylated genome, respectively. In the case of up-methylated DMGs, the BLUE and the BLUE_and_PUT treatments showed the highest impact on PC1 and PC2. The effect of PUT both at WHITE and at BLUE lights as well as the combined effect of WHITE_and_PUT have also correlated with the BLUE effects. The white light itself had the least effect on plant response under PUT treatment (Fig. 5A). Oppositely, the PCA of the pattern of the down-methylated DMGs showed, that BLUE (and the effect of PUT at BLUE illumination) correlated to the other treatments with a lesser extent, whilst the paired effect of WHITE_and_PUT and WHITE_at_PUT_effect showed the strongest correlation to each other and the impact on PCs (Fig. 5B). Consequently, the BLUE light (and PUT) may have had a significant influence on the up-methylation and white light (and PUT) on the down-methylation of the DMGs.

DMGs with the highest log_2_FC values of each light- and PUT-treatment combinations were listed in Table S7. The first ten highly methylated DMGs in the up- and down-methylated lists were rather different. Only three genes were represented in more than one treatment in the case of the down-methylated DMG list (LOC123108510—germin-like protein 8–5, LOC123068847—DEAD-box ATP-dependent RNA helicase 18-like, LOC123143509—probable pyridoxal 5′-phosphate synthase subunit PDX2) and LOC123107951—dehydrin Rab15-like, LOC123162097—BTB/POZ and MATH domain-containing protein 2-like and LOC123145097—pentatricopeptide repeat-containing protein At1g74900 were found to be common in the up-methylated list.

#### The GO enrichment analysis of the treatments

**Fig. 6:**
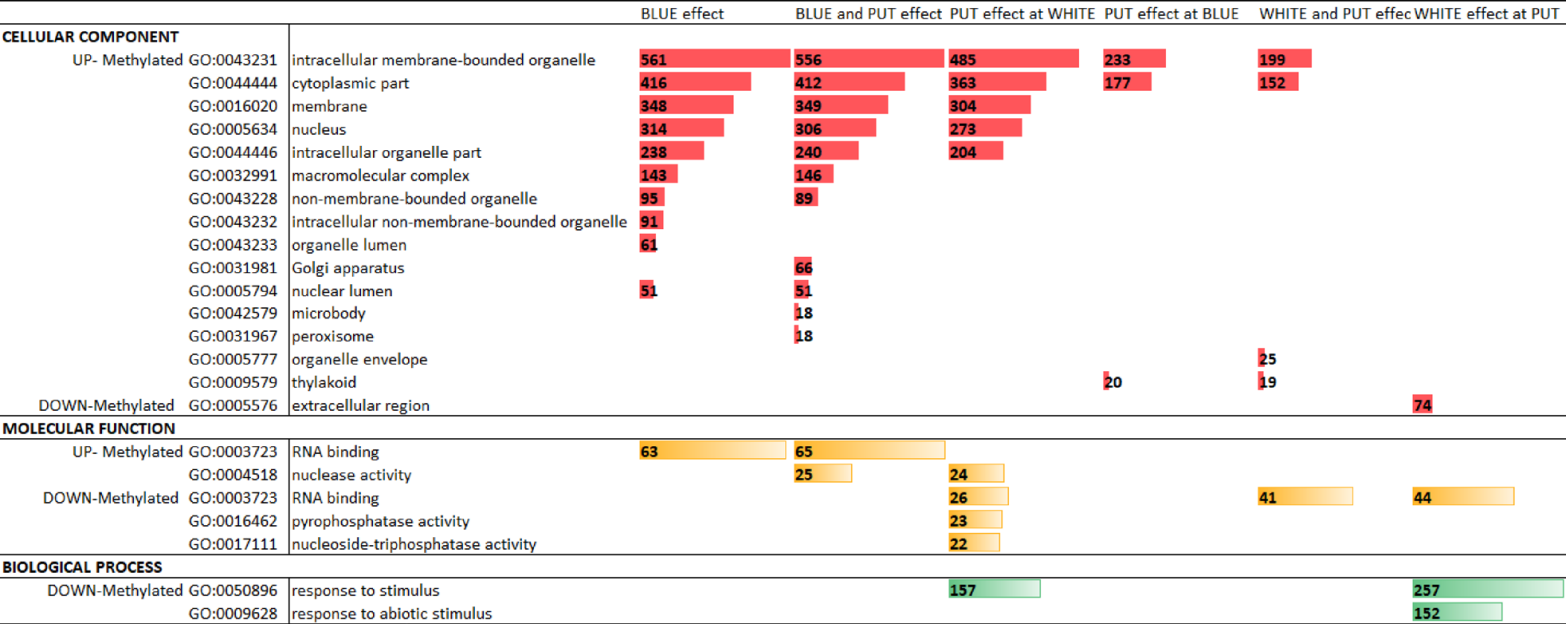
The names, IDs, and numbers of significant (FDR < 0.05) gene ontology (GO) terms in light and putrescine treatment combinations. The terms of the three major GO classes are shown: cellular component, molecular function and biological process. The numbers in the coloured bars mean the number of mapped counts compared to the background (reference). All parent and redundant terms have been truncated.

All of the DMGs belonging to the treatment combinations were investigated for biological relevance using GO enrichment analysis. The distribution of significant GO terms (FDR < 0.05) of biological process (BP), molecular function (MF) and cellular component (CC) can be seen in Fig. 6.

Of the three main GO classes, the CC was the most informative for the mapping of the DMGs to the GO terms. The up-methylated genes mostly belonged to the intracellular compartments, while the down-methylated genes were mapped to the extracellular region (WHITE effect under PUT only). Within the intracellular space, membranes or membrane-bounded organelles and the nucleus were significantly represented in large numbers under BLUE, PUT under white light, and BLUE and PUT effects. More specialized terms, such as nuclear lumen, Golgi apparatus, and peroxisome were overrepresented under the combined effect of BLUE and PUT. The thylakoid membrane was emphasized under the paired effect of WHITE and PUT, as well as the PUT effect under BLUE light.

In the case of molecular functions, up-methylated genes mapped to RNA binding and nuclease activity were the most abundant under BLUE and BLUE and PUT effects. The number of down-methylated genes showed the overrepresentation of RNA binding, nucleoside- triphosphatase- and pyrophosphatase activities under the PUT effect at white light. The RNA binding was also found to be significant under the WHITE effect under PUT and paired WHITE and PUT effects. Biological processes were represented by only a few terms from the down-methylated DMG list. Response to stimulus and response to abiotic stimulus were highlighted in the PUT effect under white light and the WHITE effect under PUT, respectively. Supplementary Table 8 contains all the unfiltered analysis results.

### Validation of gene expression profile by qRT-PCR

Based on some characteristic differences in DNA methylation (log_2_FC >|5|) between different comparisons, a few genes were selected for individual inspection. Despite the high differences at the methylation level, only slight changes were observed at the gene expression level.

**Fig. 7:**
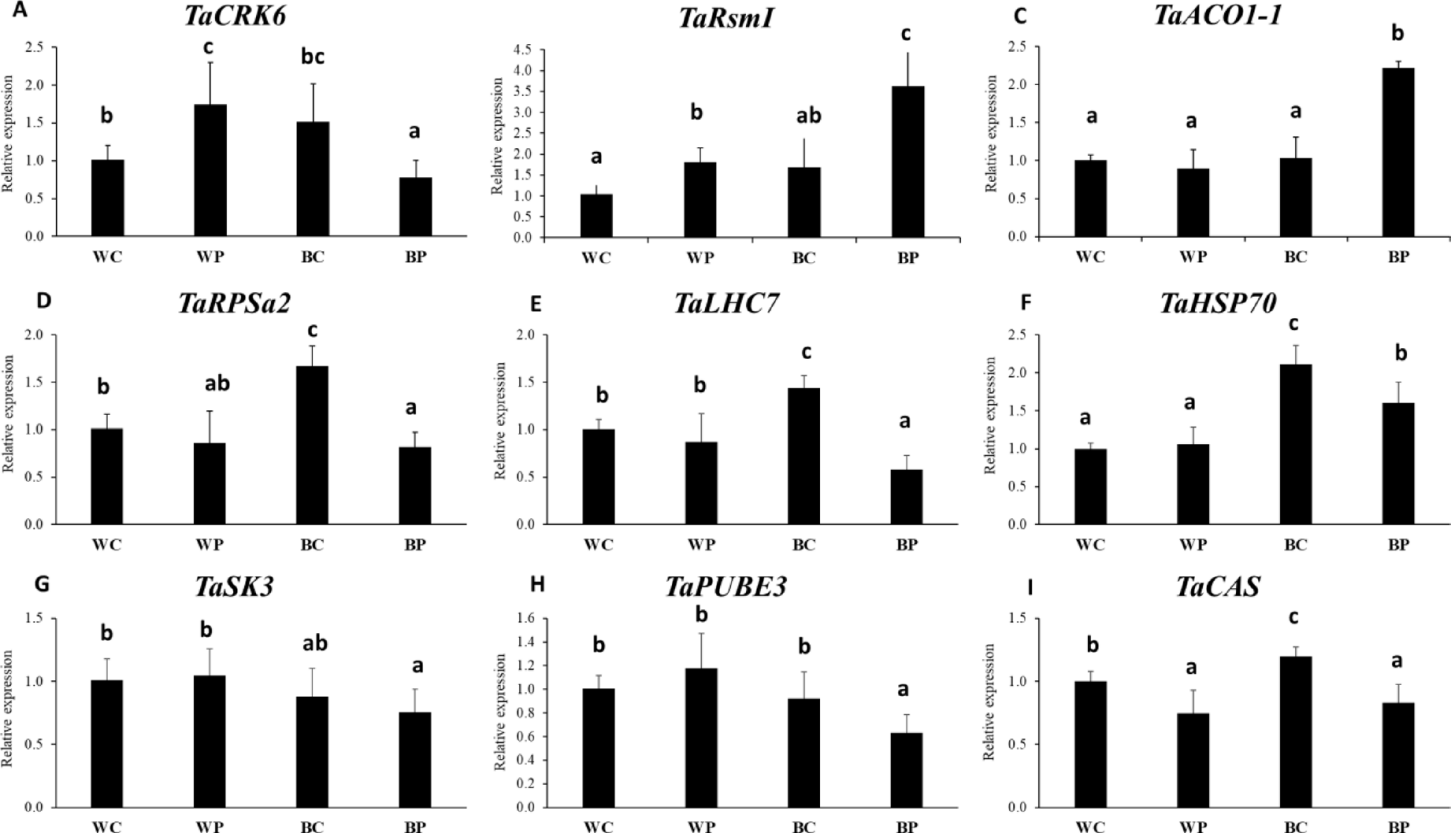
Gene expression levels of genes selected according to the most characteristics differences in DNA methylation (log_2_FC >|5|). Relative expression values were normalised for the wheat *Ta2291 – ADP-ribosylation factor*.

*TaCRK6* was down-methylated under BC compared to WC treatment, while up-methylated after BP and WP compared to BC treatment. The results of qRT-PCR showed a slight, but statistically not significant increase in *TaCRK6* expression under BC compared to WC, and a significant decrease in BP-treated plant compared to the others (Fig. 7A).

*TaRsmI* was up-methylated after BC, BP and WP treatments compared to the WC, while the transcript level of *TaRsmI* was higher after all the treatments, especially after BP compared to the WC conditions (Fig. 7B).

In the case of *TaACO1-1* also up-methylation was detected after all the treatments compared to the WC, but at the gene expression level, only BP treatment induced significantly the level of *TaACO1-1* transcript (Fig. 7C).

*TaRPSa2* was up-methylated under B light conditions either with or without PUT treatment compared to the WC treatment, in contrast, it was down-methylated after WP treatment compared to the BC or BP treatment. Neither of the differences at the methylation level manifested at gene expression level (Fig. 7D).

The *TaLHC7* gene was down-methylated by PUT treatment either under W or B light conditions compared to the adequate controls, however the gene expression pattern showed partly opposite patterns. (Fig. 7E).

*TaHSP70* was also down-methylated under B light with or without PUT treatment compared to the WC treatment, but up-methylated after PUT treatment under W light compared to BC or BP treatments. These changes were also manifested at gene expression level, as the transcript level of *TaHSP70* was indeed higher under B light conditions (BC or BP) compared to WC, and lower after WP treatment compared to BC or BP treatment (Fig. 7F).

*TaSK3* was down-methylated in WP compared to WK, BK or BP treatments, and its expression level was slightly higher in WP compared to BK or BP treatments (Fig. 7G).

Similar results were found for *TaPUBE3*, as it was also down-methylated in WP compared to WK, BP or BK, and the highest expression level of it was found in the case of the WP treatment (Fig. 7H).

*TaCAS* gene was down-methylated in BC compared to WC, but up-methylated in BP and WP compared to BC treatment. According to these, its highest expression level was found in BC-treated plants (Fig. 7I).

## Discussion

Light is an important factor that can affect not only the synthesis of functional metabolites but also induces gene expression changes in plants. B light also induces characteristic, well-documented changes in various plant species^44–47^. The capability of re-modelling plant processes at metabolite and gene expression levels allows plants to acclimate under changing environments. In turn, light can also influence the application and effects of plant growth regulators, such as PAs. The biochemical roles and effects of PAs have been demonstrated in many physiological processes, for example in the stability and function of proteins and nucleic acids, improvement of photosynthesis, regulation of N/C balance, plant growth and development and stress responses^1^. Several studies demonstrated the effectiveness of exogenous PA treatments against abiotic stresses, such as salt, drought, flooding, heavy metal or extreme temperature stress^48^. The protective effects of PAs can be explained by many changes they induce, such as increased antioxidant capacity, synthesis of protective compounds, changes in hormonal balance, and changes in the gene expression level of different stress-related genes^49,50^.

Previously, it has been reported, that less pronounced cadmium stress was detected under blue light than at white light conditions. Under blue light lower Cd stress was observed compared to the white light conditions, which was accompanied with some other differences at metabolite and gene expression levels. 7 days of 0.5 mM PUT pre-treatment had a protective effect rather under white light conditions, which was manifested especially in modulating effects on phytochelatin synthesis, PA metabolism, and accumulation of phenolic compounds and plant hormones^28^. These findings demonstrated that blue light regulated Cd tolerance in wheat and modified defence strategy at several levels, when excess PUT was present. Although PAs are hypothesised to influence on DNA methylation^51^, only a few data are available on the effects of PAs at epigenetic level. In addition, the influence of light quality, i.e. blue light has not been investigated. The present study is the first where the effect of PUT was investigated at the DNA methylation level using MeDIP-seq method in wheat plants to highlight changes in the methylation profile. Since close relationship between PA metabolism and light characteristics is exist^2^, thus PA metabolism is modulated by light quality, in the present study, both W and B light conditions were tested.

Earlier both blue light and PA treatments have been separately reported to cause changes in plant metabolomics. For example, plant growth under blue light resulted in special changes in the composition of the glutamate amino acid group^26^ or in the ratios of free amino acids in the glutamate and aspartate families^52,53^ in wheat leaves. In contrast, in a different wheat genotype blue light could not influence the amino acid content^5^. Blue-enriched light background also affected the levels of compounds related to the TCA cycle in cucumber^44^, and kohlrabi^54,55^. Changes in amino acid composition were also found after PUT treatment in wheat leaves^56^. In rice, the application of PUT had no significant effect, but SPD and SPM enhanced amino acid biosynthesis during grain filling^57^. Exogenous SPD could also promote the TCA cycle, allowing the plant to produce other metabolic precursor substances in tomato plants under high temperature stress^58^. The connection of PA metabolism not only with amino acid synthesis, but also with the TCA cycle and γ-glutamyl cycle is not surprising, as Glu produced from TCA cycle is a precursor of Orn synthesis, which is one of the main substrates for PUT synthesis, and GABA is a catabolite of PUT oxidation can enter back into the TCA cycle^59^. Thus, in the present study, the first step was to investigate the changes in plant metabolites after PUT treatment under W and B light conditions. The results of metabolic profiling enabled the distinction between light-responsive and nonresponsive metabolites, and in addition, revealed specific PUT treatment-induced changes at metabolite level. B light-induced remarkable changes were observed in certain amino acid and TCA cycle-related acid content, suggesting that the applied B light was enough sufficient to investigate the modulating effect of it. However, the PUT treatment had also some specific effects, which sometimes were the exact opposite of what was detected in B light-treated plants. In the PUT-treated plants under B light, the dominant effect of blue light prevailed only in case of aconitic acid and malic acid, while the effect of PUT in the case of Asn, Gly, shikimic acid, oxalic acid, Glp and Orn. A particular additive effect of PUT and B light was observed for Tre, Ser, Phe and Glu. (Fig. S1).

Parallel with the characteristic changes at the levels of amino acids and organic acids, PUT treatments induced an increase only in the leaf SPD content under W light conditions, but in the roots increased the PUT level under W light, and decreased the SPD content under both light conditions, which changes cannot be explained by the changes in the expression level of *TaSPDS* gene. However, in the leaves lower gene expression level of *TaSPDS* was detected under B light conditions compared to the W light conditions. Similarly, it was previously shown that B-light does not affect the endogenous PA content of leaves, but decreases the transcript level of all PA metabolism-related genes investigated^2^.

Investigation on in vitro wheat embryo culture regeneration and DNA methylation patterns revealed that 0.5 and 1.5 mM PUT induced the highest levels of genomic template stability and the SPD level correlated with DNA hypermethylation^10^. In *Pinus radiata* trees, it was found that juvenile plants were characterised by a lower DNA methylation degree but higher free PA content, while mature trees showed doubled cytosine methylation degree and lower free PA level^60^. Similarly, relationships between DNA methylation levels, free PA contents and embryogenic potentials have been reported in *Pinus nigra* Arn. cell culture^61^. SAM is a precursor for both the synthesis of higher PAs (SPD and SPM) and DNA methylation, but its metabolite linkage can result in distinct changes in gene expression. It has been reported that PA deficiency could decrease methylation in certain cases, but increased methylation in other DNA regions^14^. However, in cabbage, it was found that PUT application decreased the methylation under both control and salt stress conditions^12^. In *C. tigrine*, a negative relationship was found between the PUT level and methylation rate, too^11^.

Under the present conditions, we found that the light spectra did not induce pronounced differences in the leaf PA levels, and exogenous PUT only increased slightly the SPD content, however, specific changes were found in the pattern of up- and down-methylation of DNA. Based on the GO analysis B light alone induced only up-methylation in cellular component terms, and PUT treatment under B light did not induce remarkable further changes, indicating the dominant effect of B light. Interestingly, when WP treatment was compared to BC or BP, the methylation level was only slightly different. However, under W light, PUT treatment had a higher influence, and down-methylation also occurred in molecular function and biological processes. These results indicated that PUT treatment under W light conditions had higher and more complex effects than under B light conditions.

The PCA analysis also revealed that the B light-induced up-methylation pattern was similar to that of BP treatment, and the most distinct effect was found in the case of WP compared to the former ones. The effect of B light either with or without PUT treatments on down-methylation also was more similar to each other compared to the other treatments. However, the analysis of the ten DMGs with the highest fold-change (log_2_FC) from each treatment combination showed that despite the above-mentioned dominant B light effect over the PUT treatment, B light and PUT treatments and their combination had a distinct effect on DNA methylation, manifested in the very different pattern of the 10 most affected gene list, without high occurrence of overlap.

Whole methylome analysis revealed that genes with regulatory functions, including methyl transfer (ribosomal RNA small subunit methyltransferase I-like isoform X1; histone-lysine N-methyltransferase SUVR3-like; tRNA (adenine(58)-N(1))-methyltransferase non-catalytic subunit trm6-like isoform X1), transcription and translation (transcription factor MYBC1-like; translation initiation factor IF-2-like; transcriptional corepressor LEUNIG-like isoform X9), RNA processing (pre-mRNA splicing factor SR-like 1 isoform X1), or plant hormone synthesis and signalling related processes (1-aminocyclopropane-1-carboxylate oxidase homolog 1-like; abscisic acid receptor PYL4-like; abscisic acid 8’-hydroxylase 1-like; AP2-like ethylene-responsive transcription factor BBM1; ethylene-responsive transcription factor 5-like; gibberellin 2-beta-dioxygenase 1-like; gibberellin-regulated protein 12-like; auxin response factor 18-like), seconder metabolism related pathways (phenylalanine ammonia-lyase-like; shikimate kinase 3, chloroplastic-like; anthocyanidin reductase ((2S)-flavan-3-ol-forming)-like isoform X1; putrescine hydroxycinnamoyltransferase 3-like; spermine synthase), defense responses (peroxidase 2-like; stromal 70 kDa heat shock-related protein, chloroplastic-like) were differentially methylated by the B and W light and/or PUT. However, the gene expression analysis of certain genes selected based on the identified different methylation events showed that there was not always a close relation between the measured gene expression patterns and methylation level. Previous studies also revealed that there is not always a strong overlap between DMGs and DEGs. For example, during the investigation on light-induced anthocyanin biosynthesis in red pear, ‘DMR-mediated DEGs’ were identified as DEGs which overlapped with DMRs and were in a negative correlation with the methylation level. However, these genes contain hypomethylated or hypermethylated promoters, too^62^. In maize, light regime-dependent methylation of the metabolism of di- and tricarboxylic acids has been investigated, and it was found that the expression of some of the genes was regulated via methylation of its promoter, while others were not controlled by promoter methylation^63^. It has also been shown that DNA methylation is dispensable but essential for the proper regulation of several biological processes in *Arabidopsis*^64^.

In conclusion, it was found that at the metabolite level PUT treatment induced dedicated changes in the amount of the investigated compounds, which changes were dominant even under B light conditions compared to the B light treatment alone. However, exogenous PUT application did not influence remarkably the endogenous PA levels in the wheat leaves under either light regime. Although, also no direct relationship was found between PA content and DNA methylation, very dedicated changes were observed in the up-, and down-methylation patterns under the B light, PUT treatments and their combination compared to the W light conditions. Our results showed that under B light conditions, B light had higher effects on methylation than the PUT treatment, while under W light conditions PUT treatment induced more alteration (Fig. S2). DNA methylation is reversible and dynamic and may have different results depending on the underlying sequence and its location in the genome. Sometimes contrasting results between the gene expression and the DNA methylation indicate that other processes are also involved in the modulation of transcription. In addition, the presented results did not indicate that for example B light receptors were affected by the DNA methylation under these conditions. Since gene body methylation is not always associated with transcriptional changes, its biological significance remains unclear. The present findings may be useful in understanding the different roles of methylation during PUT treatment under W or B light conditions.

## Electronic supplementary material

Below is the link to the electronic supplementary material.

Supplementary Material 1

Supplementary Material 2

Supplementary Material 3

Supplementary Material 4

Supplementary Material 5

Supplementary Material 6

Supplementary Material 7

Supplementary Material 8

Supplementary Material 9

Supplementary Material 10

Supplementary Material 11

Supplementary Material 12

## Data Availability

The data can be found in the manuscript and supplementary materials. The data relating to methylation analyses discussed in this publication have been deposited in NCBI’s Gene Expression Omnibus and are accessible through GEO Series accession number GSE292105 (https://www.ncbi.nlm.nih.gov/geo/query/acc.cgi?acc=GSE292105). For further clarification contact the corresponding author.
