# Supplementary figures and images for: Putrescine treatment has a higher effect on 5mC DNA methylation profile of wheat leaves under white than under blue light conditions
Source: Sci Rep. 2025 Jul 2;15:22734. doi: 10.1038/s41598-025-08184-y (PMC12214681; doi:10.1038/s41598-025-08184-y)

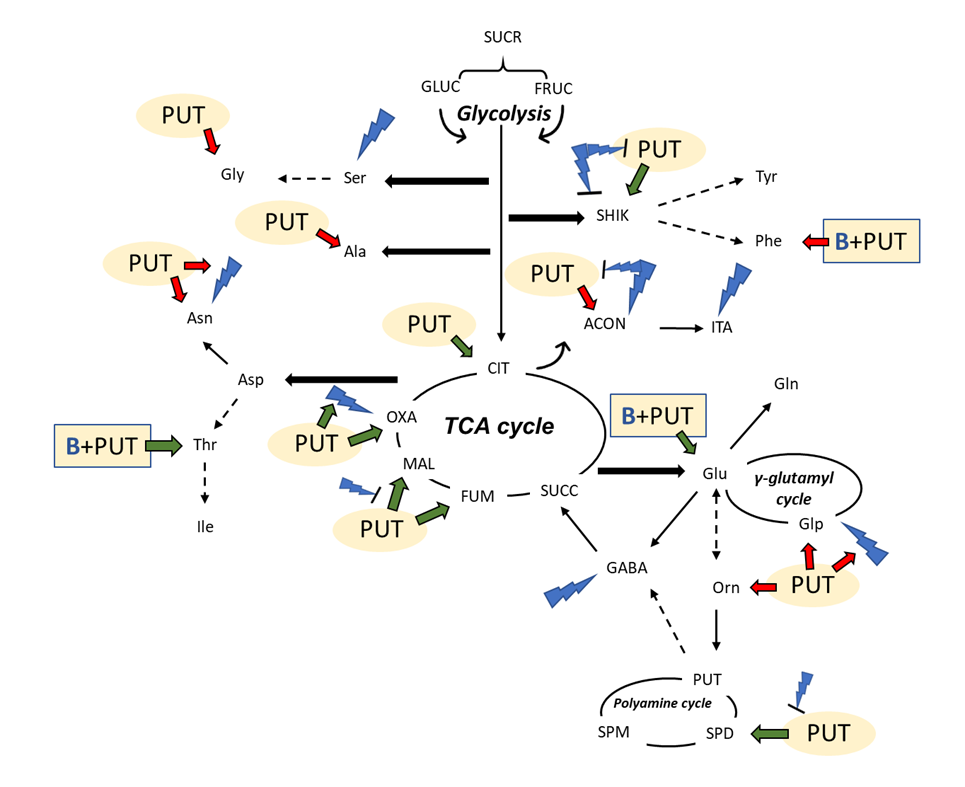

Supplement: Supplementary file 1 — Supplementary Material 1 [file 41598_2025_8184_MOESM1_ESM.tif]

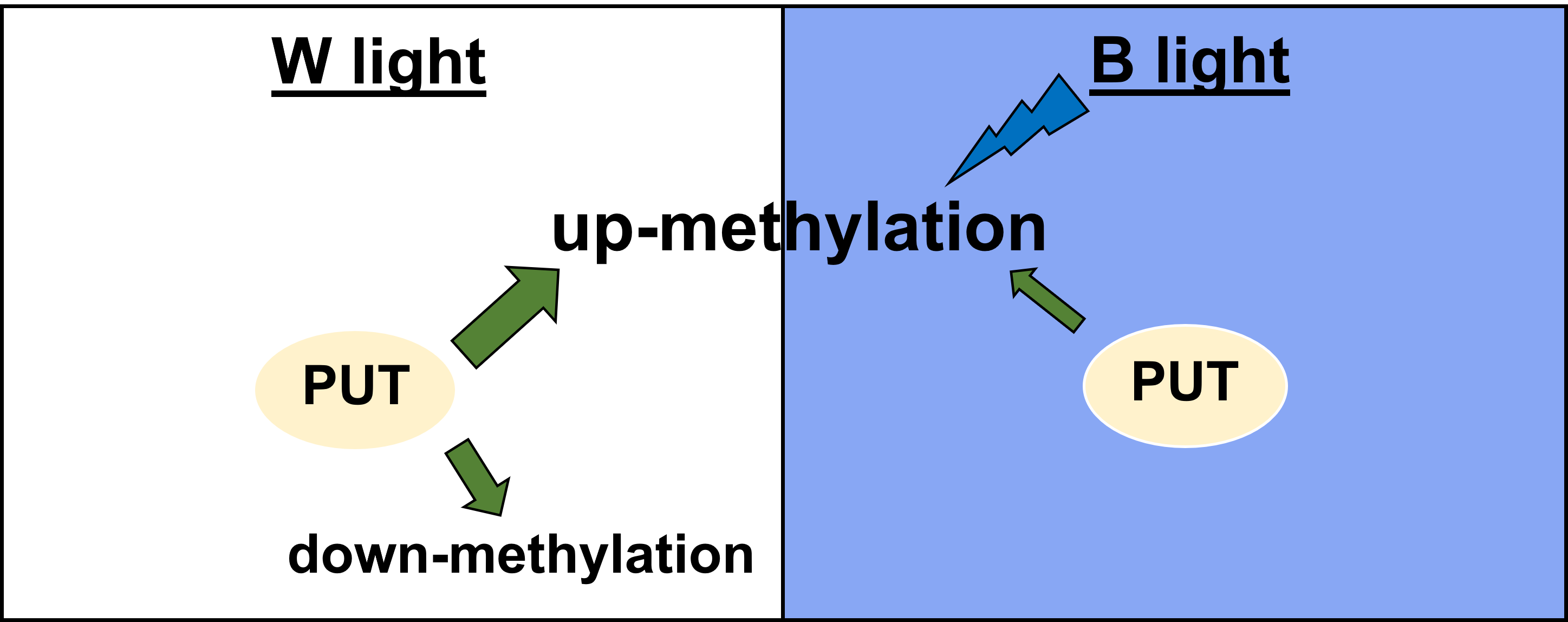

Supplement: Supplementary file 2 — Supplementary Material 2 [file 41598_2025_8184_MOESM2_ESM.tif]
