# Supplementary material for: Putrescine treatment has a higher effect on 5mC DNA methylation profile of wheat leaves under white than under blue light conditions
Source: Sci Rep. 2025 Jul 2;15:22734. doi: 10.1038/s41598-025-08184-y (PMC12214681; doi:10.1038/s41598-025-08184-y)
Supplement: Supplementary file 3 — Supplementary Material 3 [file 41598_2025_8184_MOESM3_ESM.docx]

Table 1. Characteristics of light spectral conditions.

| **Treatments** | **Intensity PAR (µmol)** | **Blue**  **µW cm^-2^ (400-500 nm)** | **Green µW cm^-2^ (500-600 nm)** | **Red µW cm^-2^ (600-700 nm)** | **Far-red µW cm^-2^ (700-800 nm)** | **Blue %** | **Green %** | **Red**  **%** | **Far-red %** | **Blue/Red** | **Red/Far-red** |
| --- | --- | --- | --- | --- | --- | --- | --- | --- | --- | --- | --- |
| **White (W)** | 250 | 1810 | 2560 | 1160 | 70 | 32.32 | 45.71 | 20.71 | 1.25 | 1.56 | 16.57 |
| **Blue (B)** | 250 | 4920 | 60 | 1000 | 10 | 82.13 | 1.00 | 16.7 | 0.17 | 4.92 | 100 |
