# Supplementary material for: Putrescine treatment has a higher effect on 5mC DNA methylation profile of wheat leaves under white than under blue light conditions
Source: Sci Rep. 2025 Jul 2;15:22734. doi: 10.1038/s41598-025-08184-y (PMC12214681; doi:10.1038/s41598-025-08184-y)
Supplement: Supplementary file 4 — Supplementary Material 4 [file 41598_2025_8184_MOESM4_ESM.docx]

Supplementary Table 2. Equations for calibration and correlation coefficients using of five different concentrations for each of the compounds. Ala: L-Alanine; ARAB: D-Arabinose; Asn: Asparagine; Asp: L-Aspartic acid; CITR: Citric acid; FUM: Fumaric acid; FRUC: D-Fructose; GABA: gamma-aminobutyric acid; Gln: L-Glutamine; Glp: L-5-Oxoproline; Glu: L-Glutamic acid; GLUC: D-Glucose; Gly: Glycine; Ile: L-Isoleucine; MAL: Malic acid; Orn: L-Ornithine; OXA: Oxalic acid; Phe: Phenylalanine; RIBI: d-Ribose; SUCC: Succinic acid; SUCR: Sucrose; Ser: Serine; Thr: L-Threonine; Tyr: L-Tyrosine.

| **Name** | **Equation for calibration** | **Correlation coefficients** |
| --- | --- | --- |
| Ala | y= +0.0551577x - 0.109023 | 0.99693 |
| ARAB | y= +0.0708314x + 0.123944 | 0.9779 |
| Asn | y= +0.00177507x - 0.00545415 | 0.99705 |
| Asp | y= +0.000335954x - 0.00119177 | 0.99574 |
| CITR | y= +0.0199898x - 0.0804283 | 0.99008 |
| FUM | y= +0.0211333x + 0.0540382 | 0.9771 |
| FRUC | y= +0.00879156x - 0.0236702 | 0.98412 |
| GABA | y= +0.0130403x + 0.0756831 | 0.99797 |
| Gln | y= +0.00859897x - 0.0189589 | 0.99348 |
| Glp | y= +0.0137944x - 0.0442122 | 0.99733 |
| Glu | y= +0.000598033x + 0.00272068 | 0.97203 |
| GLUC | y= +0.00521493x - 0.0346375 | 0.97662 |
| Gly | y= +0.00762344x - 0.0087513 | 0.99544 |
| Ile | y= +0.0230939x + 0.0970664 | 0.97869 |
| MAL | y= +0.00517747x - 0.00053731 | 0.98114 |
| Orn | y= +0.0035273x - 0.0310733 | 0.9958 |
| OXA | y= +0.0298063x - 0.0611239 | 0.98722 |
| Phe | y= +0.031772x - 0.0935744 | 0.99512 |
| RIBI | y= +0.0362438x + 0.0297867 | 0.9786 |
| SUCC | y= +0.00631735x - 0.0168699 | 0.9844 |
| SUCR | y= +0.0247896x - 0.167839 | 0.93969 |
| Ser | y= +0.0181055x + 0.0278038 | 0.98495 |
| Thr | y= +0.00649665x - 0.00139696 | 0.98977 |
| Tyr | y= +0.0248577x - 0.0367527 | 0.97936 |
