# Supplementary material for: Putrescine treatment has a higher effect on 5mC DNA methylation profile of wheat leaves under white than under blue light conditions
Source: Sci Rep. 2025 Jul 2;15:22734. doi: 10.1038/s41598-025-08184-y (PMC12214681; doi:10.1038/s41598-025-08184-y)
Supplement: Supplementary file 5 — Supplementary Material 5 [file 41598_2025_8184_MOESM5_ESM.docx]

Table S3. Primer sequences for endogenous control (*Ta*2291) and gene-specific primers.

| **Gene name and abbreviation** | **Primer sequences** | | **Product size** | **Reference** |
| --- | --- | --- | --- | --- |
| *Ta2291 –* | F | GCTCTCCAACAACATTGCCAAC | 165 bp | [42] |
| *ADP-ribosylation factor* | R | GCTTCTGCCTGTCACATACGC |  |  |
| *TaSPDS –*  *spermidine synthase* | F | AGGTATTCAAGGGTGGCGTG | 125 bp | [43] |
|  | R | TGGGTTCACAGGAGTCAGGA |  |  |
| *TaCRK6 –*  *cysteine-rich receptor-like kinase 6* | F | AGGCTGCTTGTCTACGAGTT | 101 bp | XM_044567979.1 |
|  | R | GCTTTGAGATCACGGTGGAC |  |  |
| *TaRPSa2 –*  *40S ribosomal protein Sa-2-like* | F | CCACCATTGCCTTCTGTGAC | 199 bp | XM_044599948.1 |
|  | R | TCCTCTGGGTCCCTGTAGAA |  |  |
| *TaLHC7 –*  *chlorophyll a-b binding protein 7* | F | ACCAAGATCGGCATCCTCAA | 145 bp | XM_044600141.1 |
|  | R | CGGGCTTGATGATGTCTGC |  |  |
| *TaSK3 –*  *shikimate kinase 3* | F | ACGTGCTCCCTTACCTGAAT | 145 bp | XM_044603573.1 |
|  | R | ATGCCAACAGACTGCTCAAC |  |  |
| *TaRsmI – ribosomal RNA small subunit methyltransferase I-like* | F | TGGCGATTCAAGGTACAGGT | 116 bp | XM_044466009.1 |
|  | R | TTGGCTGTCGAGATGCAAAG |  |  |
| *TaHSP70 – stromal 70 kDa heat shock-related protein* | F | TCCCTGCTCCTGTGAAAGAG | 112 bp | XM_044524175.1 |
|  | R | TCCTGGTTCAAAGCCTCCAT |  |  |
| *TaACO1 – 1-aminocyclopropane-1-carboxylate oxidase homolog 1-like* | F | AGACGTTCGGCTTCTTCCAG | 165 bp | XM_044473903.1 |
|  | R | GGTCGAGGTTGCTCTGGTAT |  |  |
| *TaPUBE3 –*  *E3 ubiquitin-protein ligase* | F | GCAGTGGTCGAGATCAGAGA | 100 bp | XM_044557678.1 |
|  | R | CAATGGACATGCGGGCTTC |  |  |
| *TaCAS –*  *calcium sensing receptor* | F | CCAACATCGTCGTCATGGAC | 161 bp | XM_044558030.1 |
|  | R | GGAGAGGTTGTAGGAGTCCG |  |  |
