# Supplementary material for: Putrescine treatment has a higher effect on 5mC DNA methylation profile of wheat leaves under white than under blue light conditions
Source: Sci Rep. 2025 Jul 2;15:22734. doi: 10.1038/s41598-025-08184-y (PMC12214681; doi:10.1038/s41598-025-08184-y)
Supplement: Supplementary file 6 — Supplementary Material 6 [file 41598_2025_8184_MOESM6_ESM.docx]

Supplementary Table 4. Variance analysis on the impact of 7 days of 0.5 mM putrescine (P) treatment under blue or white light conditions (L) on the metabolite levels in the leaves of wheat plants. (Two-way ANOVA analysis showing the mean squares, F and p-values.) ACON: Aconitic acid; Ala: L-Alanine; ARAB: D-Arabinose; Asn: Asparagine; Asp: L-Aspartic acid; CITR: Citric acid; EA: Ethanolamine; FUM: Fumaric acid; FRUC: D-Fructose; GABA: gamma-aminobutyric acid; Gln: L-Glutamine; Glp: L-5-Oxoproline; Glu: L-Glutamic acid; GLUC: D-Glucose; Gly: Glycine; Ile: L-Isoleucine; ITAC: Itaconic acid; MAL: Malic acid; Orn: L-Ornithine; OXA: Oxalic acid; Phe: Phenylalanine; RIBI: d-Ribose; SHIK: Shikimic acid; SUCC: Succinic acid; SUCR: Sucrose; Ser: Serine; Thr: L-Threonine; Tyr: L-Tyrosine. *, ** and ***: significant at 5%, 1% or 0.1 % probability level, respectively.

|  |  | **leaves** | | |
| --- | --- | --- | --- | --- |
| **Parameter** | **Source** | **MS** | **F** | ***P*** |
| **Asp** | PUT treatment (P) | 789.774 | 22.259 | **≤ 0.001 ***** |
|  | Light condition (L) | 330.755 | 9.322 | **0.009 **** |
|  | P x L | 435.888 | 12.285 | **0.004 **** |
| **Asn** | PUT treatment (P) | 1.159 | 1.710 | 0.212 |
|  | Light condition (L) | 263.424 | 388.613 | **≤ 0.001 ***** |
|  | P x L | 1.159 | 1.710 | 0.212 |
| **Thr** | PUT treatment (P) | 1403.326 | 7.943 | **0.015 *** |
|  | Light condition (L) | 1830.603 | 10.361 | **0.007 **** |
|  | P x L | 211.681 | 1.198 | 0.294 |
| **Ile** | PUT treatment (P) | 0.010 | 1.193 | 0.295 |
|  | Light condition (L) | 0.000 | 0.024 | 0.878 |
|  | P x L | 0.069 | 8.181 | **0.013 *** |
| **Ser** | PUT treatment (P) | 16310.259 | 1132.428 | **≤ 0.001 ***** |
|  | Light condition (L) | 16310.259 | 1132.428 | **≤ 0.001 ***** |
|  | P x L | 16310.259 | 1132.428 | **≤ 0.001 ***** |
| **EA** | PUT treatment (P) | 0.023 | 1.003 | 0.334 |
|  | Light condition (L) | 0.421 | 18.313 | **0.001 ***** |
|  | P x L | 0.088 | 3.832 | 0.070 |
| **Gly** | PUT treatment (P) | 220.221 | 8.631 | **0.01 **** |
|  | Light condition (L) | 68.972 | 2.703 | 0.122 |
|  | P x L | 43.021 | 1.686 | 0.215 |
| **Ala** | PUT treatment (P) | 12.289 | 25.474 | **≤ 0.001 ***** |
|  | Light condition (L) | 3.922 | 8.131 | **0.013 *** |
|  | P x L | 1.219 | 2.527 | 0.134 |
| **SHIK** | PUT treatment (P) | 78337.067 | 8.273 | **0.014 *** |
|  | Light condition (L) | 1986.626 | 0.210 | 0.655 |
|  | P x L | 40664.067 | 4.294 | 0.060 |
| **Phe** | PUT treatment (P) | 957.014 | 0.972 | 0.341 |
|  | Light condition (L) | 5912.688 | 6.006 | **0.028 *** |
|  | P x L | 1279.214 | 1.299 | 0.273 |
| **Tyr** | PUT treatment (P) | 2481.617 | 20.642 | **≤ 0.001 ***** |
|  | Light condition (L) | 7.169 | 0.060 | 0.811 |
|  | P x L | 15.526 | 0.129 | 0.725 |
| **CITR** | PUT treatment (P) | 9.243 | 5.831 | **0.033 *** |
|  | Light condition (L) | 7332.782 | 4625.874 | **≤ 0.001 ***** |
|  | P x L | 9.243 | 5.831 | **0.033 *** |
| **ACON** | PUT treatment (P) | 7680.400 | 9.644 | **0.01 *** |
|  | Light condition (L) | 1984.737 | 2.492 | 0.143 |
|  | P x L | 1933.825 | 2.428 | 0.147 |
| **ITAC** | PUT treatment (P) | 70.720 | 306.659 | **≤ 0.001 ***** |
|  | Light condition (L) | 70.080 | 303.884 | **≤ 0.001 ***** |
|  | P x L | 32.509 | 140.968 | **≤ 0.001 ***** |
| **SUCC** | PUT treatment (P) | 528.519 | 1.840 | 0.196 |
|  | Light condition (L) | 2529.630 | 8.806 | **0.011 *** |
|  | P x L | 431.577 | 1.502 | 0.241 |
| **FUM** | PUT treatment (P) | 1.151 | 4.746 | **0.047 *** |
|  | Light condition (L) | 0.271 | 1.119 | 0.308 |
|  | P x L | 1.196 | 4.931 | **0.043 *** |
| **MAL** | PUT treatment (P) | 0.094 | 0.018 | 0.895 |
|  | Light condition (L) | 180.051 | 34.780 | **≤ 0.001 ***** |
|  | P x L | 84.550 | 16.332 | **0.001 ***** |
| **OXA** | PUT treatment (P) | 2596213.711 | 25.189 | **0.001 ***** |
|  | Light condition (L) | 39024.195 | 0.379 | 0.554 |
|  | P x L | 884921.415 | 8.586 | **0.017 *** |
| **Glu** | PUT treatment (P) | 401.443 | 16.637 | **0.001 ***** |
|  | Light condition (L) | 303.721 | 12.587 | **0.003 **** |
|  | P x L | 121.220 | 5.024 | **0.042 *** |
| **Gln** | PUT treatment (P) | 1867.630 | 1.107 | 0.315 |
|  | Light condition (L) | 197.018 | 0.117 | 0.739 |
|  | P x L | 2586.338 | 1.532 | 0.242 |
| **Glp** | PUT treatment (P) | 416.395 | 0.225 | 0.643 |
|  | Light condition (L) | 1020.955 | 0.553 | 0.470 |
|  | P x L | 5724.153 | 3.100 | 0.102 |
| **Orn** | PUT treatment (P) | 6237.371 | 5.146 | **0.049 *** |
|  | Light condition (L) | 4827.550 | 3.983 | 0.077 |
|  | P x L | 0.000 | 0.000 | **≤ 0.001 ***** |
| **GABA** | PUT treatment (P) | 0.013 | 0.493 | 0.495 |
|  | Light condition (L) | 0.003 | 0.123 | 0.731 |
|  | P x L | 0.001 | 0.055 | 0.819 |
| **FRUC** | PUT treatment (P) | 0.344 | 1.378 | 0.260 |
|  | Light condition (L) | 0.718 | 2.878 | 0.112 |
|  | P x L | 0.136 | 0.547 | 0.473 |
| **GLUC** | PUT treatment (P) | 0.001 | 0.004 | 0.948 |
|  | Light condition (L) | 3.853 | 27.832 | **≤ 0.001 ***** |
|  | P x L | 0.360 | 2.604 | 0.129 |
| **ARAB** | PUT treatment (P) | 0.030 | 0.190 | 0.670 |
|  | Light condition (L) | 5.708 | 35.508 | **≤ 0.001 ***** |
|  | P x L | 1.036 | 6.446 | **0.024 *** |
| **RIBI** | PUT treatment (P) | 2.510 | 24.383 | **≤ 0.001 ***** |
|  | Light condition (L) | 0.294 | 2.856 | 0.113 |
|  | P x L | 0.004 | 0.042 | 0.841 |
| **SUCR** | PUT treatment (P) | 0.069 | 12.872 | **0.003 **** |
|  | Light condition (L) | 0.069 | 12.872 | **0.003 **** |
|  | P x L | 0.069 | 12.872 | **0.003 **** |
