# Supplementary material for: Putrescine treatment has a higher effect on 5mC DNA methylation profile of wheat leaves under white than under blue light conditions
Source: Sci Rep. 2025 Jul 2;15:22734. doi: 10.1038/s41598-025-08184-y (PMC12214681; doi:10.1038/s41598-025-08184-y)
Supplement: Supplementary file 7 — Supplementary Material 7 [file 41598_2025_8184_MOESM7_ESM.docx]

Supplementary Table 5. Variance analysis on the impact of 7 days of 0.5 mM putrescine (P) treatment under blue or white light conditions (L) on the polyamine contents in the leaves and roots of wheat plants. (Two-way ANOVA analysis showing the mean squares, F and p-values.)DAP: 1,3-diaminopropane; PUT: putrescine; SPD: spermidine; SPM: spermine. *, ** and ***: significant at 5%, 1% or 0.1 % probability level, respectively.

|  |  | **leaves** | | | | **roots** | | |
| --- | --- | --- | --- | --- | --- | --- | --- | --- |
| **Parameter** | **Source** | **MS** | **F** | ***P*** | **MS** | | **F** | ***P*** |
| **DAP** | PUT treatment (P) | 4.344 | 24.887 | **0.001 ***** | 3.875 | | 3.535 | 0.097 |
|  | Light condition (L) | 1.352 | 7.746 | **0.024 *** | 16.882 | | 15.401 | **0.004 **** |
|  | P * L | 0.450 | 2.579 | 0.147 | 0.378 | | 0.345 | 0.573 |
| **PUT** | PUT treatment (P) | 1.413 | 6.699 | **0.032 *** | 1.346 | | 2.503 | 0.152 |
|  | Light condition (L) | 0.955 | 4.526 | 0.066 | 4.724 | | 8.785 | **0.018 *** |
|  | P * L | 0.322 | 1.525 | 0.252 | 8.689 | | 16.158 | **0.004 **** |
| **CAD** | PUT treatment (P) | 0.009 | 3.529 | 0.097 | 0.011 | | 1.503 | 0.255 |
|  | Light condition (L) | 0.001 | 0.337 | 0.578 | 0.003 | | 0.390 | 0.550 |
|  | P * L | 0.035 | 13.731 | **0.006 **** | 0.015 | | 1.991 | 0.196 |
| **SPD** | PUT treatment (P) | 5.830 | 2.555 | 0.149 | 1.175 | | 0.414 | 0.538 |
|  | Light condition (L) | 18.488 | 8.103 | **0.022 *** | 104.153 | | 36.654 | **≤ 0.001 ***** |
|  | P * L | 25.711 | 11.269 | **0.01 **** | 23.171 | | 8.155 | **0.021 *** |
| **SPM** | PUT treatment (P) | 2.483 | 2.648 | 0.142 | 0.093 | | 0.590 | 0.464 |
|  | Light condition (L) | 4.283 | 4.567 | 0.065 | 5.121 | | 32.478 | **≤ 0.001 ***** |
|  | P * L | 0.021 | 0.022 | 0.886 | 1.467 | | 9.301 | **0.016 *** |
