# Supplementary material for: Putrescine treatment has a higher effect on 5mC DNA methylation profile of wheat leaves under white than under blue light conditions
Source: Sci Rep. 2025 Jul 2;15:22734. doi: 10.1038/s41598-025-08184-y (PMC12214681; doi:10.1038/s41598-025-08184-y)
Supplement: Supplementary file 11 — Supplementary Material 11 [file 41598_2025_8184_MOESM11_ESM.docx]

**Supplementary Fig. 1.** Schematic figure of the synthesis linkage between polyamines, amino acids and TCA cycle. Blue lighting icons indicate the effect of blue light. Positive or negative effects of putrescine treatment (PUT) indicated with green and red arrows, respectively. B+PUT indicates the combined effects of putrescine treatment under blue light conditions. See Table 1. for abbreviations.

**Supplementary Fig. 2.** Schematic figure for changes in methylation level induced by blue light (B) compared to white light conditions (W) with or without putrescine treatment (PUT). Blue lighting icon indicates the effect of blue light. Positive effects of putrescine treatment (PUT) indicated with green arrows; the size of the arrows reflect on the intensity of the effect. B light induced higher level of methylation compared to W light. PUT also increased the methylation level, which was more pronounced under W than under B light conditions. However, PUT also induced down-methylation in certain molecular functions and biological processes at W light.
