# Supplementary material for: Putrescine treatment has a higher effect on 5mC DNA methylation profile of wheat leaves under white than under blue light conditions
Source: Sci Rep. 2025 Jul 2;15:22734. doi: 10.1038/s41598-025-08184-y (PMC12214681; doi:10.1038/s41598-025-08184-y)
Supplement: Supplementary file 12 — Supplementary Material 12 [file 41598_2025_8184_MOESM12_ESM.docx]

**Table S6**: Overlap results between the query loci and reference genomic regions.

**Table S7**: DMGs with the highest log2FC values of each light- and PUT-treatment combinations.

**Table S8**: Unfiltered results of analysis.
